# Screening and Isolation of a Novel Polyene-Producing *Streptomyces* Strain Inhibiting Phytopathogenic Fungi in the Soil Environment

**DOI:** 10.3389/fbioe.2021.692340

**Published:** 2021-07-12

**Authors:** Heung-Soon Park, Hee-Ju Nah, Seung-Hoon Kang, Si-Sun Choi, Eung-Soo Kim

**Affiliations:** ^1^Department of Biological Sciences and Bioengineering, Inha University, Incheon, South Korea; ^2^Department of Biological Engineering, Inha University, Incheon, South Korea

**Keywords:** *Streptomyces*, phytopathogenic fungicide, polyene, BGC, genome mining, biosynthetic gene cluster

## Abstract

Microbial-based eco-friendly biological substances are needed to protect crops from phytopathogenic fungi and replace toxic chemical fungicides that cause serious environmental issues. This study screened for soil antifungal *Streptomyces* strains, which produce rich, diverse, and valuable bioactive metabolites in the soil environment. Bioassay-based antifungal screening of approximately 2,400 *Streptomyces* strains led to the isolation of 149 strains as tentative antifungal producers. One *Streptomyces* strain showing the most potent antifungal activities against *Candida albicans* and *Fusarium oxysporum* was identified as a putative anti-phytopathogenic soil isolate that is highly homologous to *Streptomyces rubrisoli* (named *S. rubrisoli* Inha 501). An *in vitro* antifungal assay, pot-test, and field-test against various phytopathogenic fungi confirmed that *S. rubrisoli* Inha 501 is a potential novel phytopathogenic fungicide producer to protect various crops in the soil environment. Whole-genome sequencing of *S. rubrisoli* Inha 501 and an anti-SMASH genome mining approach revealed an approximately 150-kb polyene biosynthetic gene cluster (BGC) in the chromosome. The target compound isolation and its BGC analysis confirmed that the giant linear polyene compound exhibiting the anti-phytopathogenic activity in *S. rubrisoli* Inha 501 was highly homologous to the previously reported compound, neotetrafibricin A. These results suggest that a bioassay-based screening of a novel antifungal *Streptomyces* strain followed by its genome mining for target compound BGC characterization would be an efficient approach to isolating a novel candidate phytopathogenic fungicide that can protect crops in the soil environment.

## Introduction

For a long time in traditional agriculture, chemical fungicides have been used to control phytopathogenic fungi, such as *Fusarium*, *Botrytis*, and *Colletotrichum*, which cause severe damage in crop production (Dean et al., 2012; Parnell et al., 2016). On the other hand, the use of chemical fungicides has been restricted because of human toxicity and ecosystem destruction (Ab Rahman et al., 2018). Alternative methods are being pursued to protect crops from phytopathogenic fungi using soil-rich microorganisms, such as *Pseudomonas*, *Bacillus*, and *Streptomyces* species, to minimize these toxic compounds in the soil environment. Several commercial products containing these microbial strains are potential biocontrol agents against various phytopathogenic fungi (Parnell et al., 2016; Ab Rahman et al., 2018; Abbasi et al., 2019). Among them is a genus called *Streptomyces*, a high G + C Gram-positive bacteria, which are ubiquitous in the soil environment. *Streptomyces* produce various useful bioactive secondary metabolites, such as antibiotics, antiviral, anticancer, anti-inflammatory, antiparasitic, and antioxidant compounds (Manivasagan et al., 2014; Jakubiec-Krzesniak et al., 2018; Bu et al., 2019). In recent years, many *Streptomyces* species present in plant roots have also shown beneficial effects on crops by controlling phytopathogenic fungi or the secretion of plant growth hormones and increasing the possibility of agricultural applications (Shi et al., 2018; Kim et al., 2019).

**FIGURE 1:**
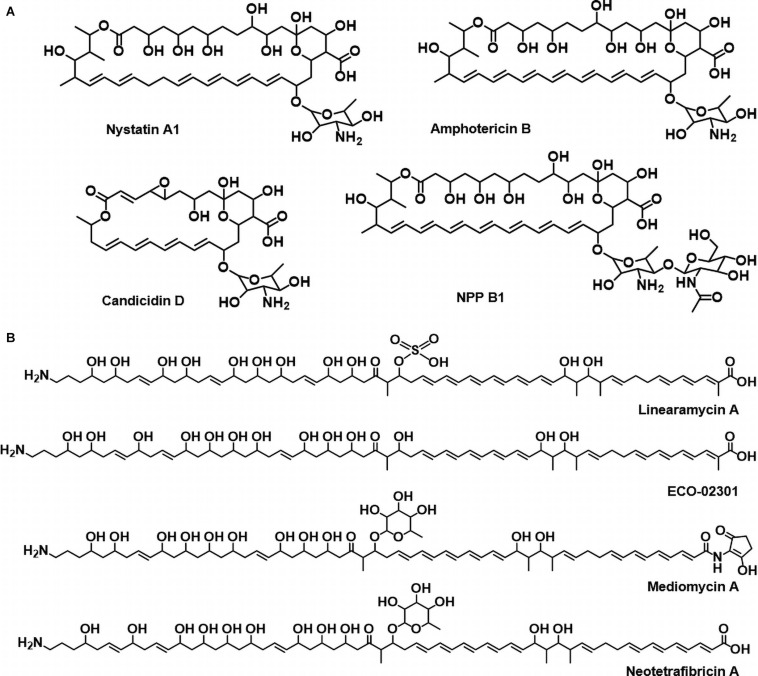
**(A)** Circular polyenes; nystatin A1, amphotericin B, candicidin D, and natamycin. **(B)** Linear polyenes; linearmycin A, ECO-02301, mediomycin A, neotetrafibricin A.

Polyene compounds typically comprise a polyketide core macrolactone ring with 20–40 carbon atoms, including 3–8 conjugated double bonds. The most characterized antifungal polyenes used primarily to treat severe fungal infections are polyene macrolides, such as the tetraene-containing nystatin A1 and heptaene-containing amphotericin B (Caffrey et al., 2016; Zhang et al., 2017). The primary antifungal mechanism of polyene antimicrobials is dependent on the interactions between the antibiotic molecules and ergosterol that appear to occur through the polyene region of the macrolactone core. In addition to these typical macrocyclic polyene compounds, there are also linear aminopolyol polyene compounds containing an amino- or guanidino-moiety, such as linearmycin, ECO-02301, mediomycin, and neotetrafibricin (Figure 1, Caffrey et al., 2016; Zhang et al., 2017). The polyene core is biosynthesized by a multi-modular giant enzyme complex called polyketide synthase (PKS), and the following processes are programmed by each domain included in PKS. In general, the acyltransferase (AT) domain selects and loads an extender unit (acyl-CoA) to the acyl-career protein (ACP) domain and the keto-synthase (KS) domain then catalyzes a decarboxylative condensation. Optionally, the keto-reductase (KR), dehydratase (DH), and enoylreductase (ER) domains reduce the β-keto group to a β-hydroxyl group, an α,β-double bond, and a saturated bond, respectively. Finally, the thioestrease (TE) domain cleaves the polyketide chain from PKS. The chain is modified by post-PKS modification enzymes, including P450 hydroxylases and glycosyltransferases (Caffrey et al., 2016; Zhang et al., 2017). The major antifungal mechanism of these polyenes is considered the formation of ion channels *via* fungal ergosterol binding that mediates the leakage of cellular K^+^ and Mg^2+^, which leads to the death of fungal cells (Neumann et al., 2016).

The traditional activity-based screening strategy is still the most widely practiced approach for selecting useful and diverse bioactive metabolites produced by soil *Streptomyces* species. Recently, alternative state-of-the-art technologies, including microbial genome mining, BGC cloning, heterologous expression, and pathway refactoring, have been pursued to complement the weaknesses of the traditional approaches, such as re-isolation and low expression issues under typical laboratory culture conditions (Lee et al., 2020). In particular, the genome mining strategy is an approach that analyzes and utilizes functional microbial genomes through bioinformatic analyses, enabling the establishment of effective strategies for predicting the pathways of various bioactive metabolite biosynthetic gene clusters (BGCs) present in the bacteria (Zerikly and Challis, 2009; Lee et al., 2020). Although a bioinformatics-based prediction using a genome mining approach still needs to be supplemented through extensive laboratory work, novel bioactive compounds can be synthesized from bacteria to various derivatives in the desired location, or natural compounds can be synthesized using sophisticated and rational reprogramming (Ziemert et al., 2016; Awakawa et al., 2018).

In this study, the bioassay-based antifungal screening of approximately 2,400 *Streptomyces* strains led to the isolation of 149 strains as tentative antifungal producers. One of these *Streptomyces* strains, showing the most potent antifungal activities against *Candida albicans* and *Fusarium oxysporum*, was identified as a putative polyene-producing soil isolate, highly homologous to *Streptomyces rubrisoli* (named *S. rubrisoli* Inha 501). An *in vitro* antifungal assay, pot-test, and field-test against various phytopathogenic fungi confirmed that *S. rubrisoli* Inha 501 could be a good candidate for a novel phytopathogenic fungicide to protect various crops in the soil environment.

## Materials and Methods

### Strains and Growth Conditions

*S. rubrisoli* Inha501 was distributed from Industrial Biomaterial Research Center, Korea Research Institute of Bioscience and Biotechnology (KRIBB), South Korea. The strain was grown routinely in ISP2 agar (malt extract 10 g, yeast extract 4 g, glucose 4 g, and agar 20 g per liter) at 30°C for the sporulation and seed culture. The R5 medium (sucrose 51.5 g yeast extract 2.5 g, peptone 5 g, malt extract 3 g, glucose 10 g, sucrose 340 g, and 10N NaOH 0.7 ml per liter) was used to produce the I-NTF (Inha-neotetrafibricin A). All *Escherichia coli* strains were incubated at 37°C in Luria–Bertani medium supplemented with the appropriate antibiotics where needed. *Candida albicans* ATCC 14053, *Aspergillus niger* ATCC 9642, *Fusarium oxysporum* f. sp. *lactucae* KACC (Korean Agricultural Culture Collection) 42795, *Fusarium oxysporum* f. sp. *gladioli* KACC 40051, *Fusarium solani* KACC 44891, *Fusarium graminearum* KACC 47495, *Fusarium verticillioides* KCTC (Korean Collection for Type Cultures) 6065, *Fusarium semitectum* KCTC 16672, *Botrytis cinerea* KACC 40574, *Colletotrichum gloeosporioides* KACC 40003, *Curvularia lunata* KACC 40861, and *Alternaria alternata* KACC 40019 were grown on PDA medium (potato starch 4 g, glucose 2 g, agar 15 g per liter) at 28°C for 2–7 days.

### Antifungal Pot-Test and Field-Test

The fermentation TSB broth of *S. rubrisoli* Inha 501 was tested for *in vivo* antifungal activity against Fusarium wilt. In the case of the pot-test, eight of each red pepper, strawberry, and tomato seedlings were prepared, respectively. The prepared seedlings were grown for 2 weeks in vinyl pots (diameter 10 cm) in a growth room at 22 ± 1°C. Four seedlings of tree crops were treated with 100 ml of *S. rubrisoli* Inha501 culture medium (10^6^ CFU/ml) along with a negative control treated only with 100 ml of TSB. After 24 h, the potted seedlings were wounded on the stem and then treated with *F. oxysporum* KACC 40051. After 2 weeks, the appearance of the seedlings was examined to measure the degree of Fusarium wilt infection (0: disease-free ∼ 100: crop failure). The pot-to-pot significance difference test was performed at the 95% level by the DMRT method (Kramer, 1957). In the case of field tests, tests were conducted in accordance with “the pesticide registration test standards and methods” in Korea (Rural Development Administration, 2008). In the evaluation, the minimum incidence rate in the untreated seedlings was more than 10%, and the effectiveness was assessed by comparing the morbidity rate with the untreated seedlings 10 days after the final *S. rubrisoli* Inha501 treatment. As for the placement of the test seedlings, the test was carried out in three repetitions of the randomized complete block design and the complete randomized design (Zerbe, 1979; Scott and Milliken, 1993). Two red pepper and tomato greenhouses each infected with Fusarium wilt were prepared. The prepared seedlings were grown in soil (10 m^2^) in a growth room at 29 ± 3°C for 4 weeks. The test seedlings were treated with 10 L of *S. rubrisoli* Inha501 culture medium (2 × 10^3^ CFU/ml) and the untreated seedlings were treated with 10 L of TSB. The pot-to-pot significance difference test was performed at the 95% level by the DMRT method (Kramer, 1957). The more detailed results of antifungal pot-test and field-test are provided as Supplementary Figure 2.

### Genome Sequencing and Assembly of *S. rubrisoli* Inha 501

The *S. rubrisoli* Inha501 genome was sequenced at Macrogen (Korea) using both the PacBio RSII (Pacific Biosciences, United States) and Illumina HiSeq (Illumina, United States) platforms. The library preparation for Illumina and PacBio sequencing was performed using the TruSeq DNA sample prep kit for Illumina (NE, United States) and the PacBio DNA Template Prep Kit 1.0 (Pacific Biosciences, United States), respectively. The library insert sizes were 350 bp for Illumina sequencing and 20 kb for PacBio RS SMRT sequencing. The *de novo* assembly of sequenced fragments was performed using Canu (v1.7) software. A high-quality sequence was obtained by performing an error correction of the assembled contig using Pilon (v1.21) software. The annotation was performed using Prokka (v1.12b) software.

### Production and Purification of Polyene Compound

The *S. rubrisoli* Inha501 was inoculated in 200 ml of TSB medium at 30°C and 220 rpm for 48 h. The pre-cultures were added to 2 L of R5 medium in a 5 L bioreactor for batch fermentation. After 120 h of cultivation, the culture broth was extracted in 2 L of *n*-butanol. The extract was concentrated using a vacuum evaporator. The concentrated extract was dissolved in methanol and loaded onto a column packed with a C18 reversed-phase silica gel (Daiso, Japan) and eluted with methanol-water (30:70, v/v) to remove any residual sugar from the production media. The extracts with the sugar removed were purified using a fraction collector (Interchim, France) on a gradient comprised of solvents A (water) and B (methanol): 30% B (v/v) (0–10 min) and 100% B (v/v) (100 min) at a flow rate of 20 ml/min. The fractions that contained I-NTF with > 90% purity were detected at 332 nm and analyzed by high-performance liquid chromatography (HPLC). The column was equilibrated with 60% solvent A (0.05 M ammonium acetate, pH 6.5) and 40% solvent B (acetonitrile). The flow rate was set to 0.5 ml/min under the following conditions: 0–30 min, 40% B.

### LC-MS/MS Analysis

The polyene compound showing > 90% purity was analyzed using A Triple TOF 5,600 + (AB Sciex, United States) coupled with Ultimate3000 (Thermo Scientific, United States). Mass spectrometry was conducted in both positive and negative ion modes over a mass range from *m*/*z* 50 to 2,000 using an electrospray ionization source. The settings were nitrogen gas for nebulization at 50 psi, heater gas pressure at 50 psi, curtain gas at 25 psi, temperature of 500°C, and an ion spray voltage at 5,500 V in positive ion mode and −4,500 V in negative ion mode. The optimized declustering potential (DP) and collision energy (CE) were set to 60 and 10 eV in positive ion mode, and −60 and −10 eV in negative ion mode, respectively. A sweeping collision energy setting at 35/−35 eV ± 15 eV was applied for collision-induced dissociation (CID). For the chromatographic conditions, solutions A (0.1% formic acid in distilled water) and B (0.1% formic acid in acetonitrile) were used for elution and loaded onto a Phenomenex Kinetex 1.7 μ C18 (2.1 mm × 150 mm, 1.7 μm). The flow rate was set to 0.4 ml/min under the following conditions: 0–1 min, 90% A; 1–5 min, 90–50% A; 5–18 min, 50–0% A; 18–25 min, 0% A; 25–27 min, 0–90% A; and 27–30 min, 90% A.

### Inactivation of I-NTF PKS I Gene

An *I-NTF PKS I* gene inactivation cassette, including homologous region of *I-NTF PKS I* gene, was constructed by PCR amplification using the following primer pairs: DELL_F (5′-GGCCA GTGCCAAGCTTCCTCGACGGGATCCGCGT-3′) and DELL_R (5′- ACATGATTACGAA TTGATCGCACTACC ACGAACCA −3′) (Supplementary Figure 5). The amplified fragments were ligated into pKC1132 digested with *Hin*dIII using an In-Fusion Cloning kit (Takara Bio, Japan). The *I-NTF PKS I* gene inactivation cassette was then introduced into *E. coli* ET12567/pUZ8007 and conjugated directly with *S. rubrisoli* Inha501 by homologous recombination. The desired mutant was selected on apramycin-included ISP2 agar medium, and its genotypes were verified by PCR.

### *In vitro* Assays for Biological Activities

The Clinical and Laboratory Standards Institute document M27-A3 was adapted to the *in vitro* antifungal assay (Clinical and Laboratory Standards Institute, 2008). After fungus was cultured in PDB medium at 30°C for 1∼7 days, the cultured solution was diluted with PDB medium until the OD value reached 0.3 at 530 nm. A working suspension was prepared by a 1:2,000 dilution with RPMI-1640 broth media (with glutamine and phenol red, without bicarbonate, Sigma-Aldrich, United States), which resulted in 5.0 × 10^2^ to 2.5 × 10^3^ cells per μl. Ten microliter of the DMSO containing polyene antibiotics at various concentrations (25–1,600 μg/ml) were added to the working suspension of 990 μl and the mixtures were then incubated at 30°C without shaking for 2∼7 days. The colorimetric change in the mixture from red to yellow indicated the growth of the fungus. The minimum inhibitory concentration (MIC) was determined by measuring the minimum concentration that changed the color to yellow. The experiment was performed in duplicate.

## Results

### Screening and Isolation of an Antifungal Soli *Streptomyces* Species

**FIGURE 2:**
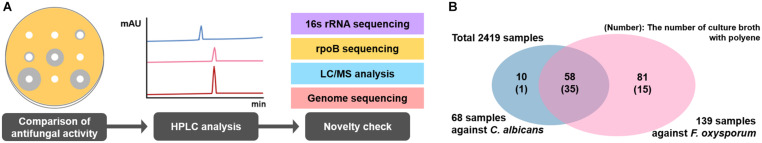
**(A)** Antifungal bioassay-based screening scheme. **(B)** Venn diagram showing the number of strains showing antifungal activities against *C. albicans* and *F. oxysporum.*

**FIGURE 3:**
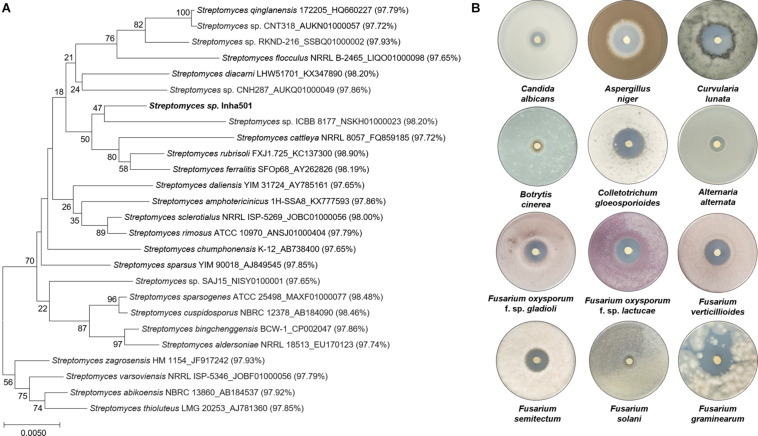
**(A)** Phylogenetic tree of *S. rubrisoli* Inha501 based on the 16s rRNA sequence; the evolutionary history was inferred using the Neighbor-Joining method. Evolutionary analyses were conducted in MEGA7 (Kumar et al., 2016). **(B)** Antifungal assays of *S. rubrisoli* Inha501 against various phytopathogenic fungi.

A total of 2,419 *Streptomyces* culture extracts provided from the actinomycetes cell collection center in the Industrial Biomaterial Research Center, Korea Research Institute of Bioscience and Biotechnology (KRIBB), Korea was screened to isolate the strains showing antifungal activities against *C. albicans* (a fungal strain for the confirmation of antifungal activity) and *F. oxysporum* (a phytopathogenic fungal strain) (Figure 2 and Supplementary Figure 1). 149 antifungal culture extracts exhibiting antifungal activity were first selected, followed by the additional selection of 51 antifungal strains showing a typical polyene spectrum assayed by 2-dimensional HPLC analysis (Figure 2, Guo et al., 2000). Among the 51 strains tested, one *Streptomyces* sp. isolated from organic green tea fields in Jeolla Province in Korea (34°51′31.7^″^N 127°08′48.1^″^E) was selected as a final candidate to control phytopathogenic fungi, showing the strongest antifungal activities against *C. albicans* and *F. oxysporum*. 16s rRNA sequence-based phylogenetic analysis showed that the strain isolated above exhibited 98.9% similarity of *S. rubrisoli* FXJ1.725 (KC137300) and was called *S. rubrisoli* Inha501 (Figure 3A).

Laboratory-scale antifungal bioassays were performed to assess the potential applications for the development of phytopathogenic fungicide using the isolated *S. rubrisoli* Inha501 against various phytopathogenic fungi, *F. oxysporum, F. solani*, *F. graminearum*, *F. verticilliodes*, *F. semitectum*, *Aspergillus niger*, *Botrytis cinerea, Colletotrichum gloeosporioides*, *Curvularia lunata*, and *Alternaria alternata*. As shown in Figure 3B, *S. rubrisoli* Inha501 showed a strong and broad spectrum of phytopathogenic antifungal activities against most phytopathogenic fungi tested.

The controllability of phytopathogenic fungi in several crops was tested by performing pot-scale tests with the *S. rubrisoli* Inha501 culture in the plants infected with *F. oxysporum* (KACC 40051). As shown in Table 1A, the fungal pathogen control rate for each red pepper, strawberry, and tomato was observed at 70.6, 69.2, and 63.2%, respectively. Moreover, the fungal pathogen control rate in the field-scale test was 56.3 and 58.2% for the naturally infected red pepper and tomato, respectively, implying a positive outlook for its registration as a biocontrol microbial agent (Table 1B and Supplementary Figure 2).

**TABLE 1:** *In vivo* antifungal activity of *S. rubrisoli* Inha501 against Fusarium wilt.

(A) Pot-test against *F. oxysporum.*									

Pot test	Tomato^a^			Strawberry^b^			Red pepper^c^		
	Disease incidence (%)	DMRT	Control rate (%)	Disease incidence (%)	DMRT	Control rate (%)	Disease Incidence (%)	DMRT	Control rate (%)
Inha501	35.0	bc	63.2	15.0	b	69.2	23.1	bc	70.6
No-treatment	95.0	a	–	48.8	a	–	78.8	a	–

*Coefficient of variation – ^*a*^ 13.0%, ^*b*^21.2%, ^*c*^19.3%*									

**(B) Field-test against *Fusarium wilt***							

**Field test I^a^**	**Tomato**			**Field test II^b^**	**Tomato**		
	**Disease incidence (%)**	**DMRT**	**Control rate (%)**		**Disease incidence (%)**	**DMRT**	**Control rate (%)**

Inha501	5.3	b	65.0	Inha501	6.3	b	51.5
No-treatment	15.4	a	–	No-treatment	13.1	a	–

**Field test III^c^**	**Red pepper**			**Field test IV^d^**	**Red pepper**		
	**Disease incidence (%)**	**DMRT**	**Control rate (%)**		**Disease incidence (%)**	**DMRT**	**Control rate (%)**

Inha501	5.1	b	57.5	Inha501	10.5	b	55.1
No-treatment	12.0	a	–	No-treatment	23.14	a	–		

*Coefficient of variation – ^*a*^15.5%, ^*b*^10.7%, ^*c*^17.9%, ^*d*^29.4%.*

### Whole-Genome Sequencing and Characterization of Biosynthetic Gene Clusters

**FIGURE 4:**
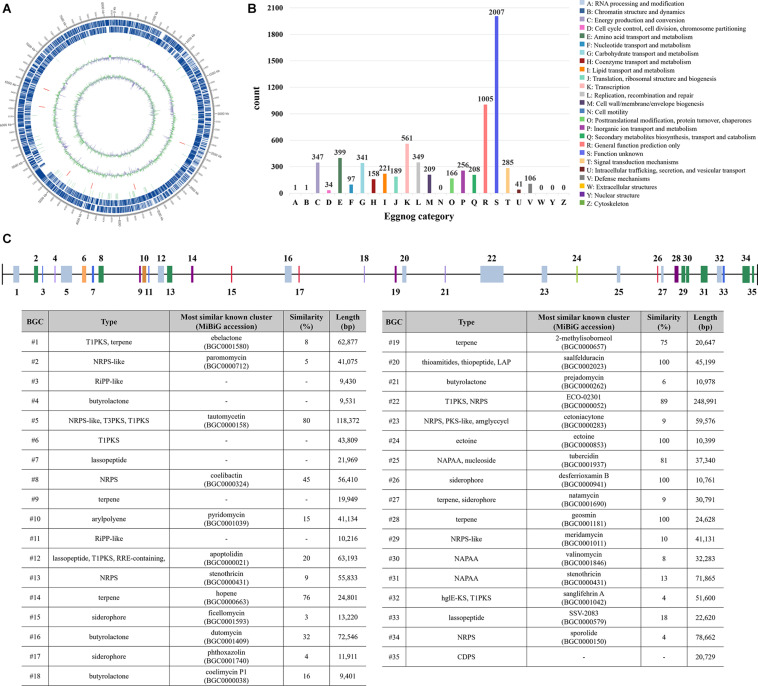
**(A)** Circular whole-genome map drawn by applying the annotation result of the *S. rubrisoli* Inha501 chromosome. Marked characteristics are shown from the outside to the center; CDS on the forward strand, CDS on the reverse strand, tRNA, rRNA, GC content, and GC skew **(B)** Classification of CDS by eggnog annotation **(C)** Analysis of secondary metabolite biosynthetic gene clusters by antiSMASH 5.0.

Whole-genome sequencing was performed to identify the biosynthetic gene cluster (BGC) present in the *S. rubrisoli* Inha501 chromosome, which is responsible for producing the target antifungal compound. The complete genome size was 8,249,972 bp, and the G + C content was 70.19% (Figure 4A and Supplementary Table 1). The whole genome sequence was deposited in Strategic Initiative for Microbiomes in Agriculture and Food^1^ with an accession number igem-0000408. The *S. rubrisoli* Inha501 genome contained 2 plasmids (85,489, 32,253 bp), 7,318 genes, 75 tRNAs, and 18 rRNAs (Supplementary Table 1). The encoding gene sequences were aligned with the eggnog databases to predict the putative gene functions (Huerta-Cepas et al., 2019). Among them, 6,881 genes were successfully annotated with Eggnog, accounting for 94% of all genes (Figure 4B).

The antiSMASH 5.0 program revealed 35 tentative BGCs of the secondary metabolites present in the *S. rubrisoli* Inha501 chromosome (Blin et al., 2019). BGC # 22 was predicted to be the most likely BGC to synthesize the target linear polyene compound (Figure 4C). Bioinformatic analysis of BGC #22 predicted the typical biosynthetic pathway of the target polyene compound and revealed a significant similarity to the BGC of the previously reported neotetrafibricin A (NTF A) (Table 2 and Supplementary Figure 3). LC-MS analysis of the purified target polyene compound (>90% purity) in the *S. rubrisoli* Inha501 culture showed a signal at m/z 1226.73 (calculated mass, 1227.73), which is the same as the signal at m/z 1226.72 for [C_6__7_H_10__4_NO_19_]^–^ of NTF A (Zhang et al., 2017; Supplementary Figure 4).

**TABLE 2:** Annotation of the ORFs in the I-NTF gene cluster.

ORF	Size^a^	Predicted function	*S. aizunensis* and *S. neyagawaensis* homolog^b^	Accession number
I-NTF A	234	Thioesterase	ORF1 (65/75%)	AAX98176.1
I-NTF B	920	Transcriptional regulator	ORF2 (55/65%)	AAX98177.1
I-NTF C	210	Response regulator	ORF3 (75/84%)	AAX98178.1
I-NTF D	417	Sensor kinase	ORF4 (65/76%)	AAX98179.1
I-NTF E	182	Putative membrane protein	ORF5 (64/75%)	AAX98180.1
I-NTF F	163	Putative membrane protein	ORF6 (82/89%)	AAX98181.1
I-NTF G	520	Putative membrane protein	ORF7 (63/75%)	AAX98182.1
I-NTF H	373	Glycosyltransferase	ORF8 (74/82%)	AAX98183.1
I-NTF PKS I	9792	Type I PKS	Tfb1 (72%/80%)	BAW35651.1
I-NTF PKS II	9298	Type I PKS	Tfb2 (68/76%)	BAW35652.1
I-NTF PKS III	6493	Type I PKS	Tfb3 (72/79%)	BAW35653.1
I-NTF PKS IV	1633	Type I PKS	Tfb4 (74/83%)	BAW35654.1
I-NTF PKS V	5121	Type I PKS	Tfb5 (74/81%)	BAW35655.1
I-NTF PKS VI	5309	Type I PKS	Tfb6 (71/79%)	BAW35656.1
I-NTF PKS VII	3169	Type I PKS	Tfb7 (78/85%)	BAW35657.1
I-NTF PKS VIII	7352	Type I PKS	Tfb8 (75/82%)	BAW35658.1
I-NTF PKS IX	3755	Type I PKS	Tfb9 (74/81%)	BAW35659.1
I-NTF I	317	Acyltransferase	ORF18 (66/75%)	AAX98193.1
I-NTF J	298	Putative membrane protein	ORF19 (65/76%)	AAX98194.1
I-NTF K	349	ABC transporter	ORF20 (68/79%)	AAX98195.1
I-NTF L	335	Sugar dehydratase/epimerase	ORF21 (58/67%)	AAX98196.1
I-NTFM	210	Sugar epimerase	ORF22 (69/82%)	AAX98197.1
I-NTF N	355	Sugar nucleotidyltransferase	ORF23 (74/81%)	AAX98198.1
I-NTF O	328	Sugar dehydratase/epimerase	ORF24 (77/82%)	AAX98199.1
I-NTF P	227	Thioesterase	ORF25 (55/66%)	AAX98200.1
I-NTF Q	467	Acyl CoA ligase	ORF26 (78/87%)	AAX98201.1
I-NTF R	544	Amine oxidase	ORF27 (76/83%)	AAX98202.1
I-NTF S	226	Phosphopantetheinyl transferase	ORF28 (56/67%)	AAX98203.1
I-NTF T	303	Metallophosphoesterase	ORF29 (80/84%)	AAX98204.1
I-NTF U	952	Transcriptional regulator	ORF30 (51/64%)	AAX98205.1
I-NTF V	533	Carboxylase/carboxyltransferase	ORF31 (86/91%)	AAX98206.1
I-NTF W	332	Amidinohydrolase	ORF32 (79/90%)	AAX98207.1

*^*a*^Size in the number of amino acids.*

*^*b*^S. aizunensis NRRL B-11277 is the production strain of ECO-02301 (ORF 1∼32) (McAlpine et al., 2005). S. neyagawaensis NR0557 is the production strain of neotetrafibricin A (Tfb 1∼9) (Zhang et al., 2017). The homolog shows amino acid sequence identity%/similarity%.*

**FIGURE 5:**
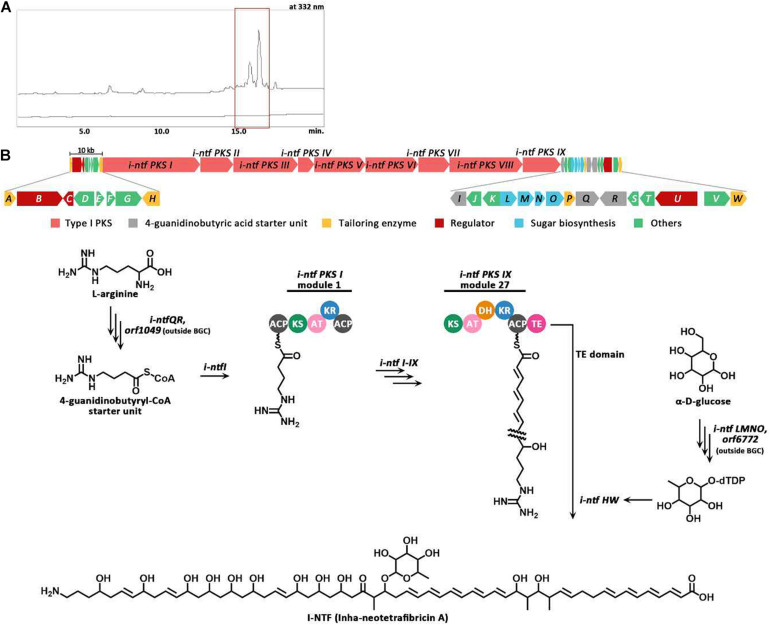
**(A)** HPLC chromatograms of the cultures from the wild-type *S. rubrisoli* Inha501 (upper line) and the *i-ntf PKS I* disrupted *S. rubrisoli* Inha501 mutant (lower line). The asterisked peak is confirmed to be the I-NTF and the small peak in front of I-NTF peak is believed to the derivative or degradation product of I-NTF **(B)** ORFs encoded in the putative I-NTF biosynthetic gene cluster and proposed biosynthetic pathway to I-NTF (more details of ORFs are listed in Table 2).

The PKS gene (Inha501-4694) of BGC #22 was disrupted in the *S. rubrisoli* Inha501 chromosome to confirm that BGC #22 is responsible for biosynthesis of the NTF A-like compound (Figure 5A and Supplementary Figure 5). As expected, the BGC #22 PKS gene-disrupted mutant strain failed to produce NTF A-like compound, suggesting that the BGC #22 is indeed responsible for the biosynthesis of NTF A-like compound in *S. rubrisoli* Inha501 (tentatively called Inha-neotetrafibricin A, I-NTF) (Yoo et al., 2011; Hong et al., 2013; Zhang et al., 2017; Figure 5B and Supplementary Figure 6).

### Biological Activities of I-NTF

The purified I-NTF was evaluated for its antifungal activity by *in vitro* assays using a paper disc. The antifungal activity of I-NTF was confirmed in *C. albicans* and 11 phytopathogenic fungi. The size of the inhibition zone was larger than the controls, Amphotericin B and Nystatin A1 (Supplementary Figure 7). The antifungal activity was measured by examining the MIC (minimum inhibitory concentration) evaluation assays of the purified I-NTF using the colorimetric change in the RPMI-1640 media containing the fungus (Clinical and Laboratory Standards Institute, 2008; Supplementary Figure 8). The MIC of I-NTF against *C. albicans*, *A. niger*, and *F. oxysporum* was lower than nystatin A1 but higher than that of amphotericin B (Table 3). Interestingly, the MIC value of I-NTF against *F. verticillioides*, *F. semitectum*, *C. lunata*, and *A. alternata* was lower than nystatin A1 and amphotericin B (Table 3). In summary, the antifungal activity of I-NTF produced by *S. rubrisoli* Inha501 against 12 fungi (containing 11 phytopathogenic fungi) was confirmed.

**TABLE 3:** *In vitro* antifungal activity.

Antifungal activity (MIC^*a*^, μg/ml)			

Strain	Amphotericin B	Nystatin A1	I-NTF
*C. albicans* ATCC 14053	0.5	4	2
*A. niger* ATCC 9642	0.5	4	2
*F. oxysporum* KACC 40051	2	8	2
*F. oxysporum* KACC 42795	1	8	4
*F. verticillioides* KCTC 6065	1	8	<0.25
*F. semitetum* KCTC 16672	1	4	0.5
*A. alternata* KACC 40019	>16	>16	4
*C. lunata* KACC 40861	16	>16	4
*C. gloeosporioides* KACC 40003	0.5	2	4
*B. cinerea* KACC 40574	1	2	16

*^*a*^MIC, minimum inhibitory concentration (values resulting in no visible growth of the fungus).*

## Discussion

In place of synthetic fungicides containing various environmental and health issues, considerable research for the isolation and application of eco-friendly microorganisms has been conducted. In this study, activity-based screening allowed the isolation of 149 strains of antifungal *Streptomyces* that exhibited antifungal activity out of the 2,419 strains tested. Existing activity-based screening strategies had limitations that the type and chemical structure of target compounds produced from selected strains were unknown until accurate structural analysis was completed. PCR screening approaches using the primers specific to polyene specific P450 genes were also implemented to select the novel strain producing a specific structural family, such as polyene compounds (Hwang et al., 2007). Complete genome sequencing of the selected strain complemented the weakness of the activity-based strategy, and the BGC analysis made it feasible to predict the tentative structure of the target compound.

Through the whole genome sequence analysis of *S. rubrisoli* Inha501, 35 putative BGCs were predicted, of which seven BGCs contained the PKS genes as the core biosynthetic genes. Thanks to the co-linearity nature of the sequence, the biosynthetic pathway of the type I PKS system and its structure are relatively easy to predict in accordance with the usual rules of the PKS multi-domain modular system. Through anti-SMASH analysis of all BGCs present in the *S. rubrisoli* Inha501 strains, it was predicted that the target antifungal compound encoded by the BGC #22 was the previously reported giant linear polyene compound called NTF A (Zhang et al., 2017). The compound produced by *S. rubrisoli* Inha501 was tentatively named I-NTF (Inha neotetrafibricin) until a more definite structure analysis was completed. The gene-knockout experiments confirmed that BGC #22 is responsible for the biosynthesis of an I-NTF. Interestingly, I-NTF biosynthetic gene knockout showed a decrease in overall antifungal activity in *S. rubrisoli* Inha501, but the activity did not disappear completely, suggesting that there might be additional functional antifungal BGCs in *S. rubrisoli* Inha501, such as BGC #5 (80% similarity to tautomycetin) and BGC #25 (81% similarity to tubercidin) (Choi et al., 2017; Liu et al., 2018).

The newly isolated *S. rubrisoli* Inha501 confirmed its antifungal activity against various phytopathogenic fungal strains and showed its potential applications as a bio-control agent through pot-test and field-test for actual crops, such as strawberries, tomatoes, and red peppers. Optimization of the culture condition and its formulation study is currently being pursued for the development of microbial fungicides of *S. rubrisoli* Inha501. The synergistic approach between activity-based screening and genome analysis highlights the efficient isolation of novel strains, such as *S. rubrisoli* Inha501, which contains a giant linear polyene BGC and a strong anti-phytopathogenic compound to protect various crops growing in the soil environments.

## Data Availability Statement

The datasets presented in this article are not readily available because the government-funded whole genome sequence is deposited in the local members-only genome database. Requests to access the datasets should be directed to eungsoo@inha.ac.kr.

## Author Contributions

H-SP, S-SC, and E-SK designed the experiments. H-SP, H-JN, and S-HK performed the experiments. H-SP and E-SK wrote the manuscript. All authors contributed to the article and approved the submitted version.

## Conflict of Interest

The authors declare that the research was conducted in the absence of any commercial or financial relationships that could be construed as a potential conflict of interest.

## Footnotes

1 http://igem.or.kr
