# Supplementary material for: Screening and Isolation of a Novel Polyene-Producing Streptomyces Strain Inhibiting Phytopathogenic Fungi in the Soil Environment
Source: Front Bioeng Biotechnol. 2021 Jul 12;9:692340. doi: 10.3389/fbioe.2021.692340 (PMC8312574; doi:10.3389/fbioe.2021.692340)
Supplement: Supplementary file 1 [file Data_Sheet_1.docx]

**Screening and isolation of a novel polyene-producing *Streptomyces* strain inhibiting phytopathogenic fungi in soil environment.**

**Heung-Soon Park**^1^**, Hee-Ju Nah**^1^**, Seung-Hoon Kang**^1^**, Si-Sun Choi**^1^**,**

**and Eung-Soo Kim**^1, 2,^**^*^**

^1^Department of Biological Sciences and Bioengineering, Inha University, Incheon 22212, Korea

^2^Department of Biological Engineering, Inha University, Incheon 22212, Korea

^*^Corresponding authors:

E-mail: eungsoo@inha.ac.kr; Phone: +82-32-860-8318; Fax: +82-32-865-4046

Key words: *streptomyces,* phytopathogenic fungicide, polyene, BGC, genome mining

**Supplementary Figure S1.** Screening to isolate the strains showing both antifungal activities against *C. albicans* and *F. oxysporum* and a typical polyene spectrum assayed by 2-dimensional HPLC analysis. **(A)** antifungal assay against *C. albicans* and *F. oxysporum* **(B)** HPLC analysis

**
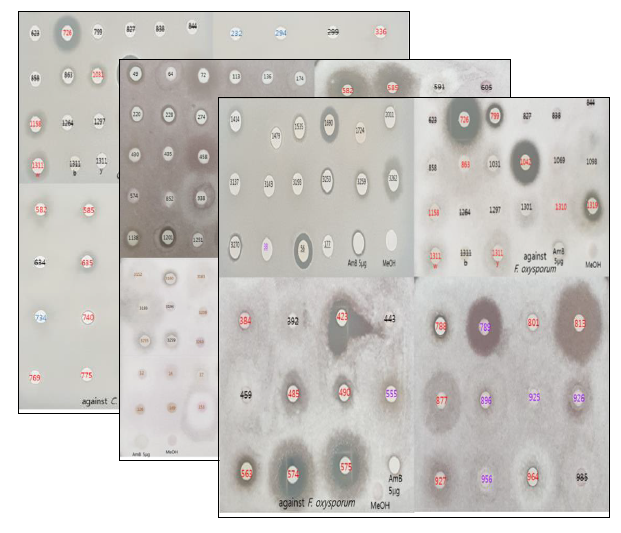
A.**

**
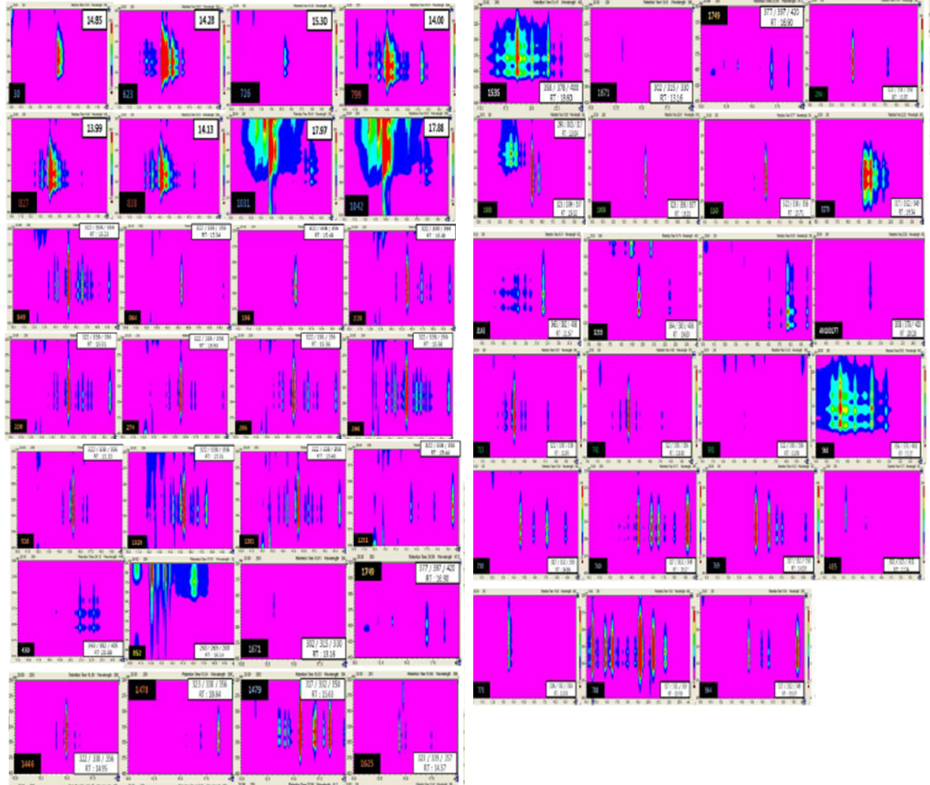
B.**

**Supplementary Figure S2.** **(A)** Antifungal pot-test **(B)** Antifungal field-test


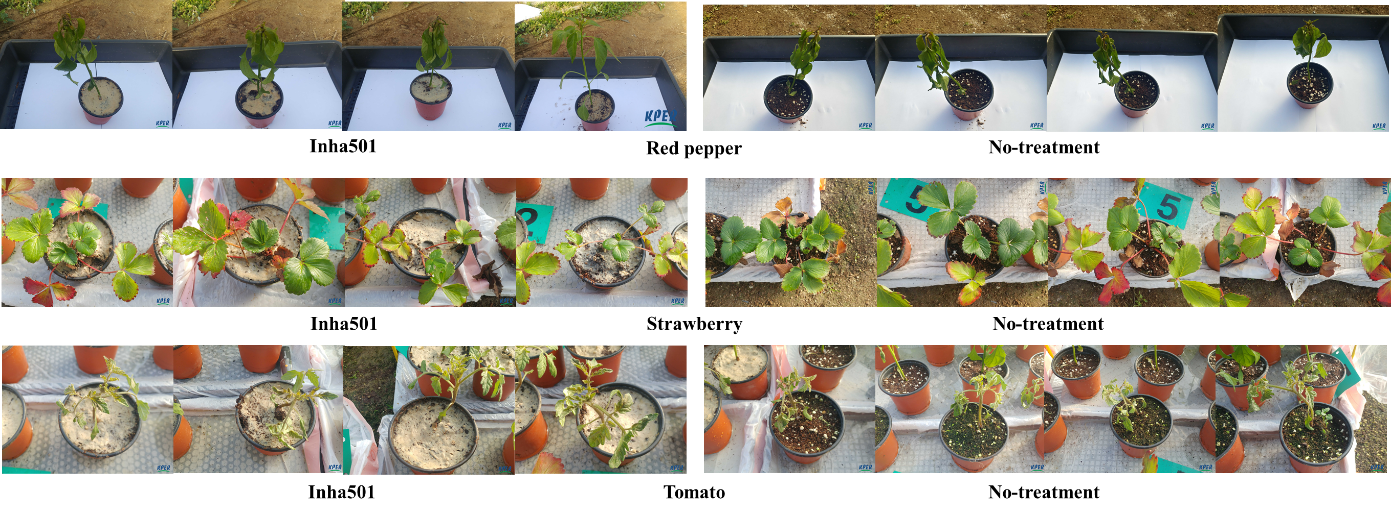
A.


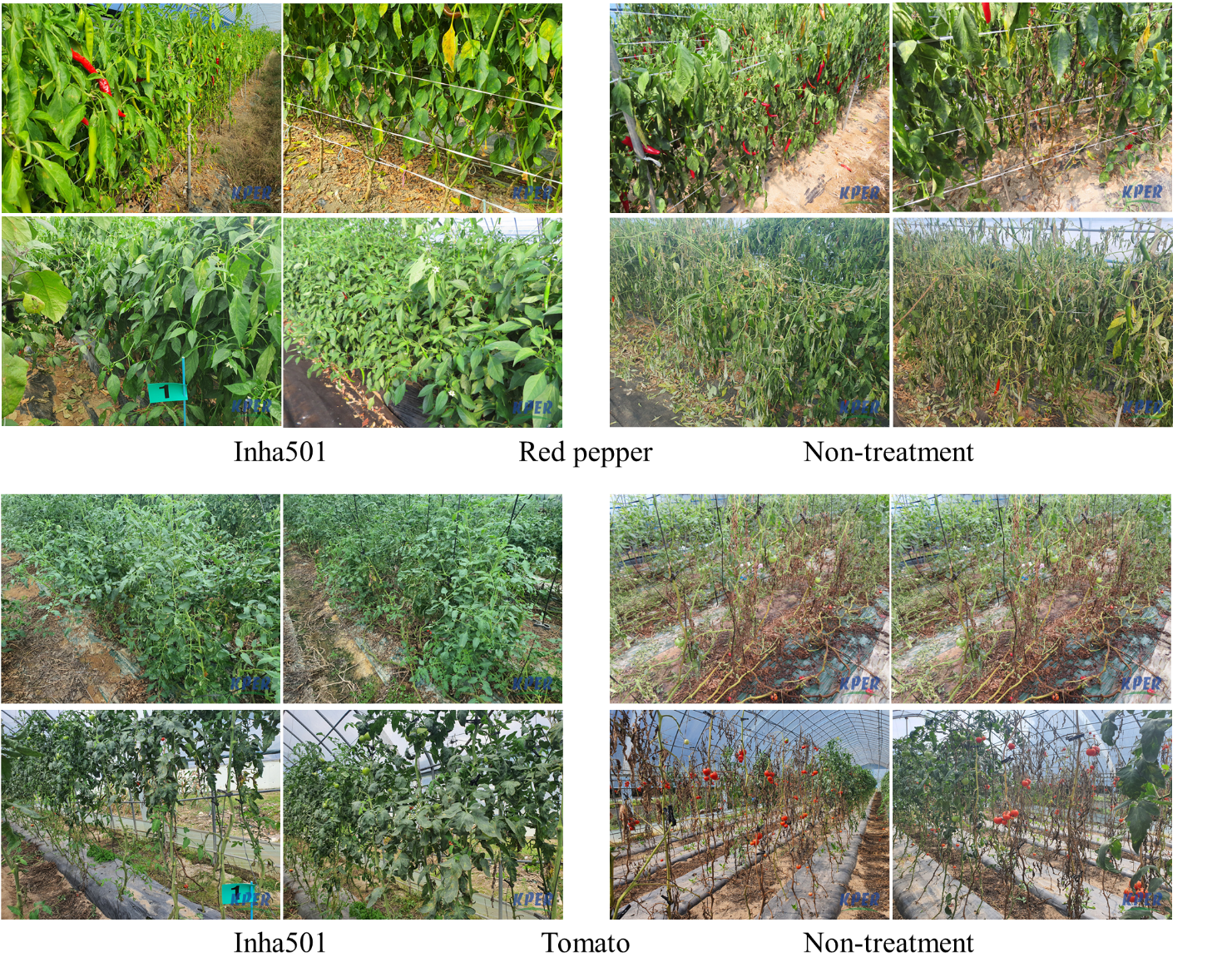
B.

**Supplementary Figure S3*.*** Comparison of PKS gene in I-NTF and NTF A BGC (by antiSMASH).


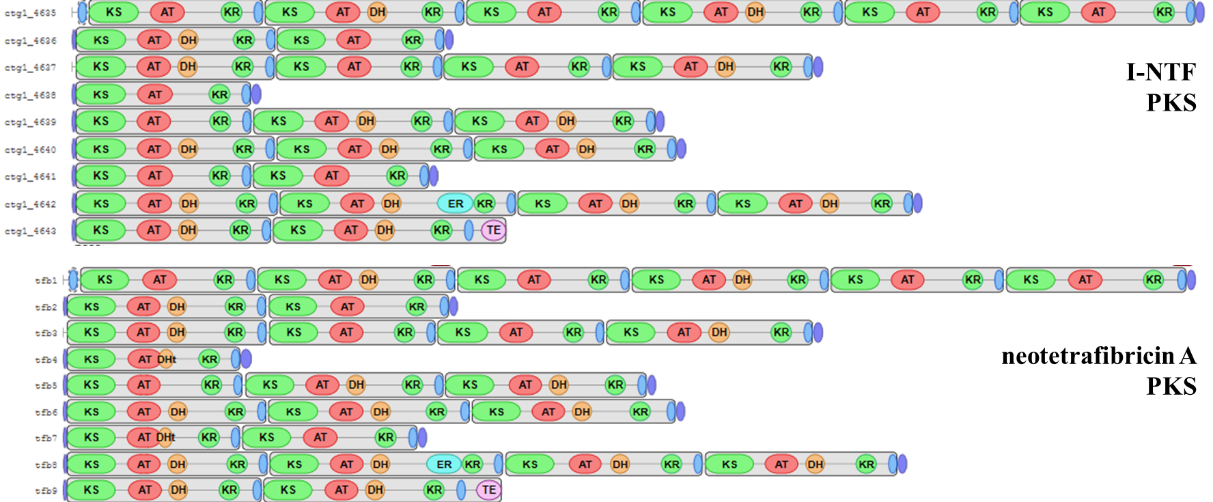


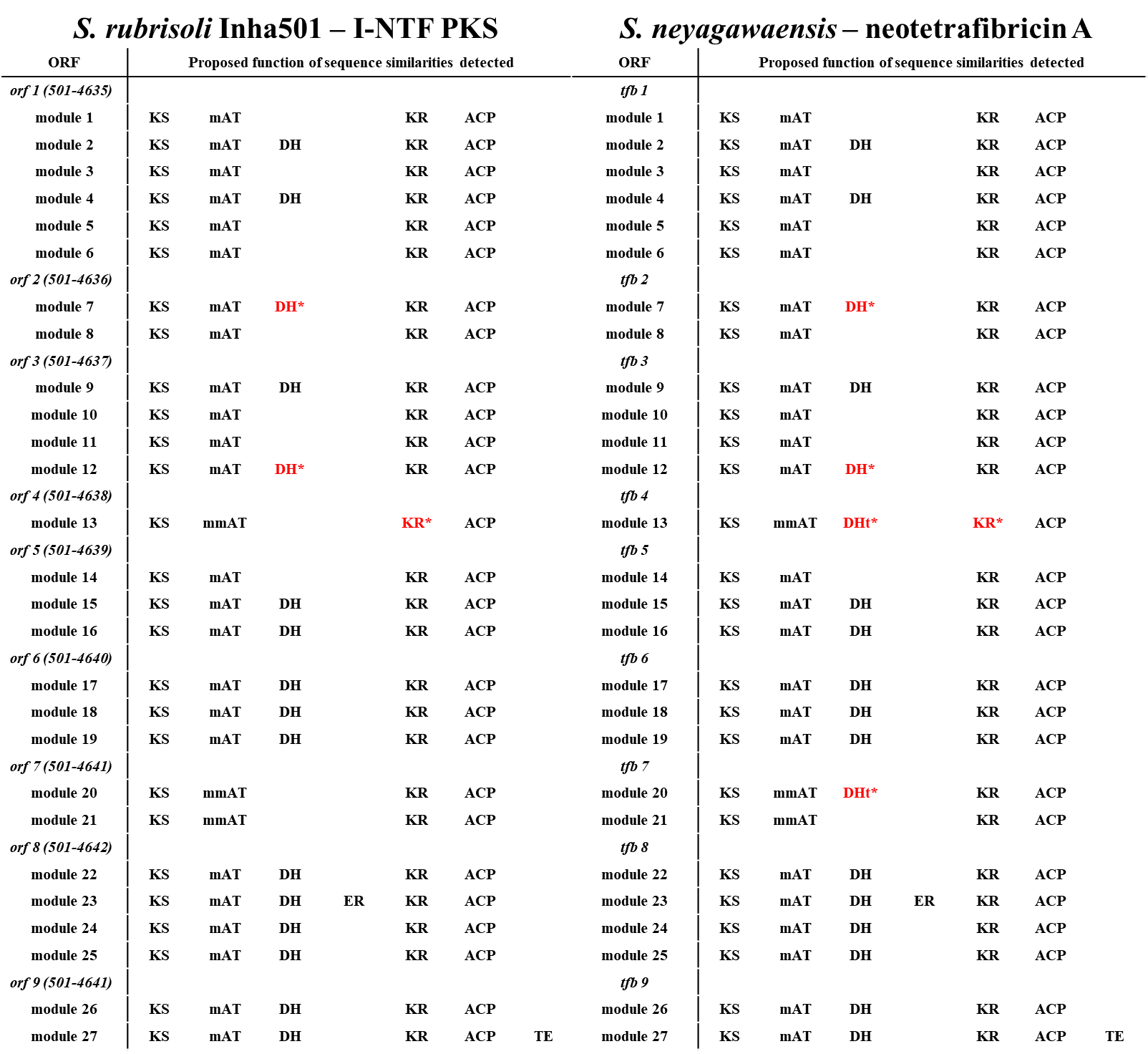


**Supplementary Figure S4.** High Resolution Mass Spectrometry (HRMS) in negative ion mode to measure the molecular weight of I-NTF.


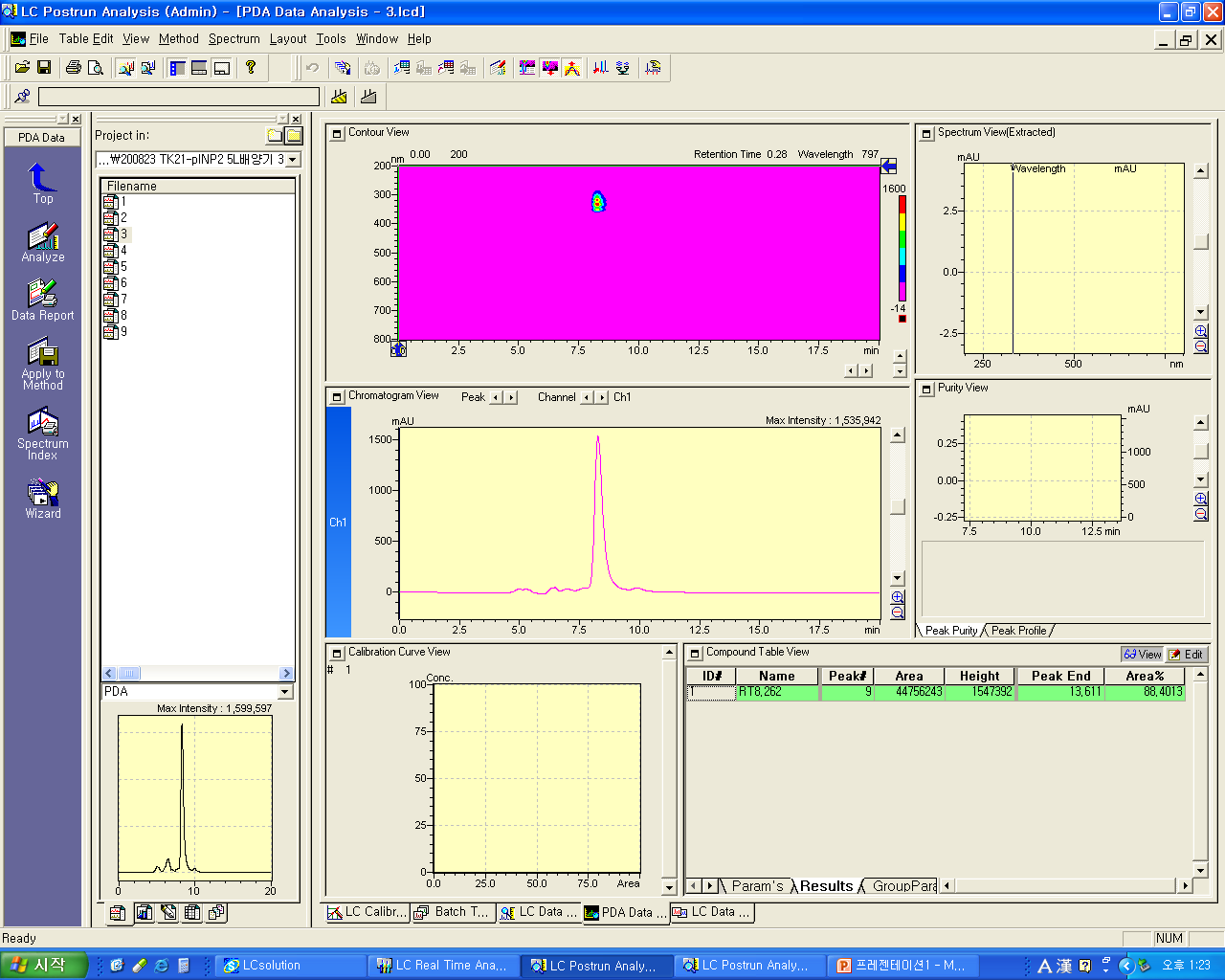

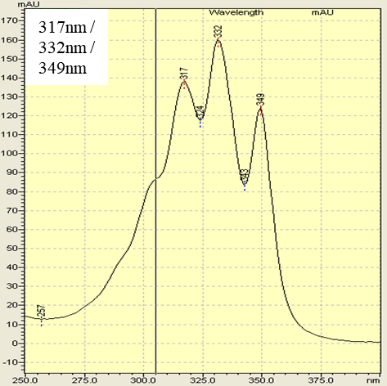


I-NTF

MS

I-NTF

I-NTF

MS/MS

**Supplementary Figure S5.** Confirmation of I-NTF BGC by gene knockout. (Check primer pair; F (5’-TTGTAAAACGACGGCCAGTG-3’) and R (5’- AAGACCGTTACTCGCACCAT-3’)


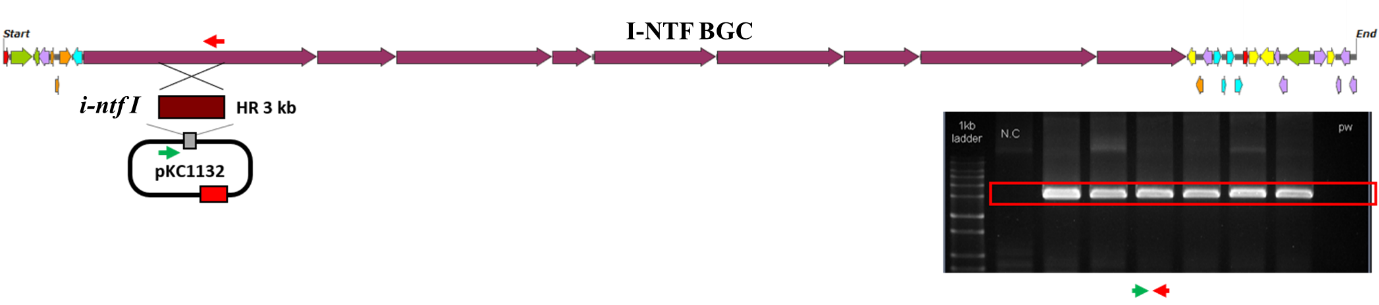


**Supplementary Figure S6.** Proposed I-NTF biosynthetic pathway in *S. rubrisoli* Inha501.


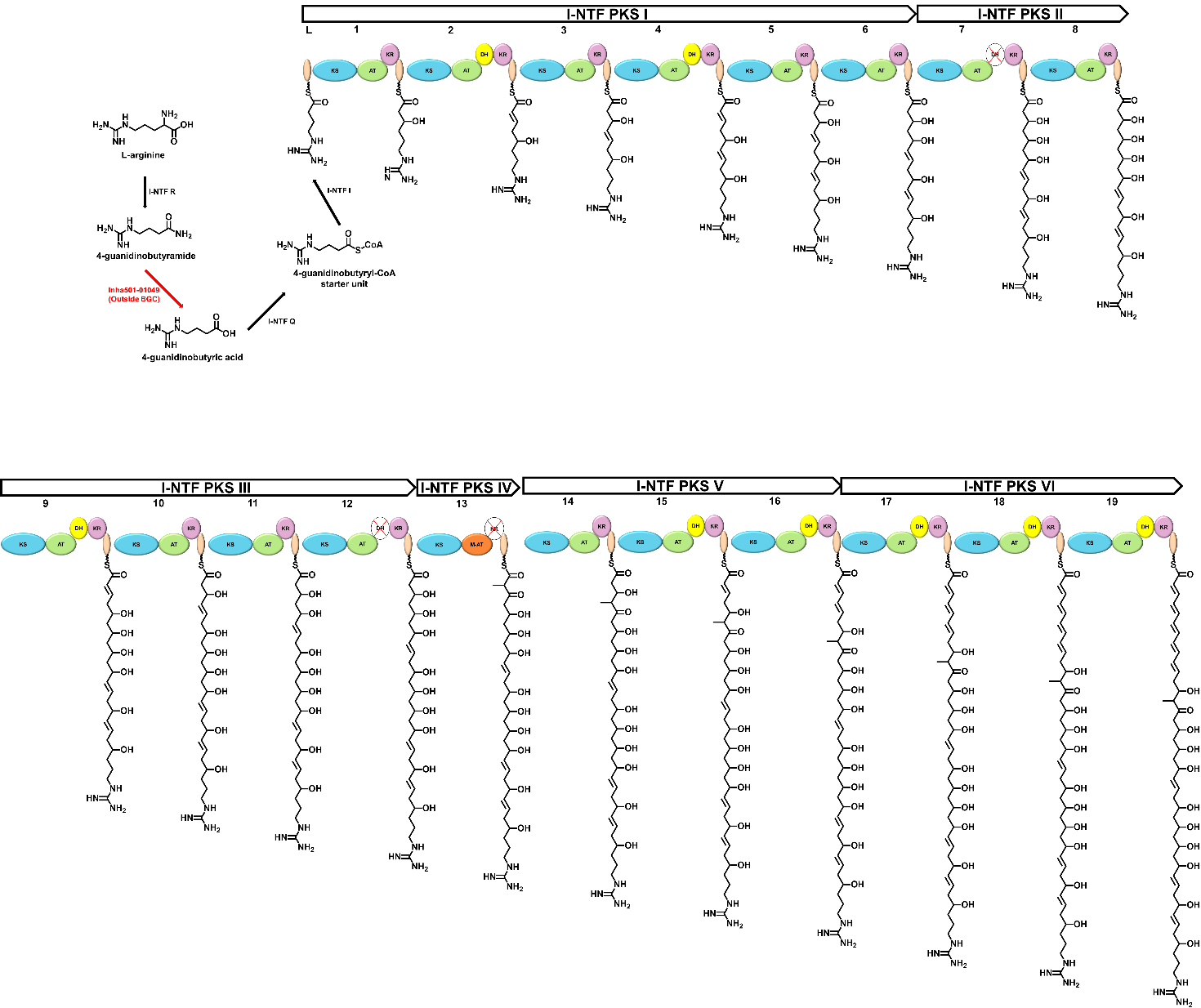


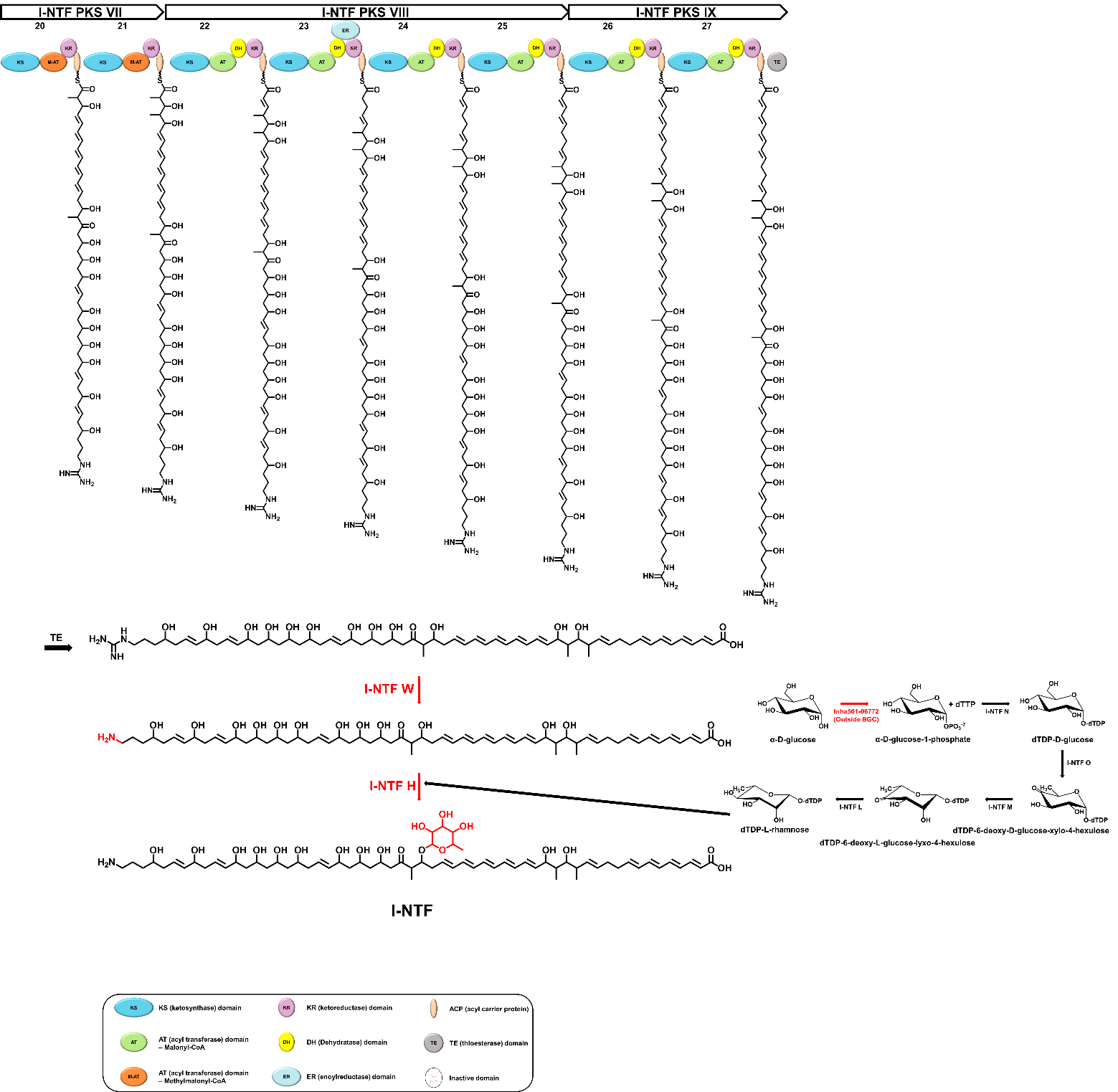


**Supplementary Figure S7.** antifungal activity of I-NTF against *C. albicans* and 11 phytopathogenic fungi.

**KS**

**M-AT**

**KR**

**I-NTF PKS IV**

**13**

**KS**

**AT**

**KR**

**KS**

**AT**

**DH**

**KR**

**KS**

**AT**

**KR**

**KS**

**AT**

**DH**

**KR**

**I-NTF PKS III**

**9**

**10**

**11**

**12**

**KS**

**AT**

**DH**

**KR**

**KS**

**AT**

**KR**

**I-NTF PKS II**

**7**

**8**

**KS**

**AT**

**KR**

**KS**

**AT**

**DH**

**KR**

**KS**

**AT**

**KR**

**KS**

**AT**

**DH**

**KR**

**KS**

**AT**

**KR**

**KS**

**AT**

**KR**

**I-NTF PKS I**

**L**

**1**

**2**

**3**

**4**

**5**

**6**

**KS**

**AT**

**DH**

**KR**

**KS**

**AT**

**DH**

**KR**

**KS**

**AT**

**DH**

**KR**

**I-NTF PKS VI**

**17**

**18**

**19**

**KS**

**AT**

**DH**

**KR**

**KS**

**AT**

**KR**

**KS**

**AT**

**DH**

**KR**

**I-NTF PKS V**

**14**

**15**

**16**

**KS**

**AT**

**DH**

**KR**

**KS**

**AT**

**DH**

**KR**

**KS**

**AT**

**DH**

**KR**

**KS**

**AT**

**DH**

**KR**

**ER**

**I-NTF PKS VIII**

**22**

**23**

**24**

**25**

**KS**

**AT**

**DH**

**KR**

**KS**

**AT**

**DH**

**KR**

**TE**

**I-NTF PKS IX**

**26**

**27**

**KS**

**M-AT**

**KR**

**KS**

**M-AT**

**KR**

**I-NTF PKS VII**

**20**

**21**

**I-NTF W**

**I-NTF H**

**I-NTF**

**I-NTF R**

**Inha501-01049**

**(Outside BGC)**

**I-NTF Q**

**I-NTF I**

**L-arginine**

**4-guanidinobutyramide**

**4-guanidinobutyric acid**

**4-guanidinobutyryl-CoA**

**starter unit**

**+ dTTP**

**Inha501-06772**

**(Outside BGC)**

**I-NTF O**

**I-NTF N**

**I-NTF M**

**I-NTF L**

**α-D-glucose**

**α-D-glucose-1-phosphate**

**dTDP-D-glucose**

**dTDP-6-deoxy-D-glucose-xylo-4-hexulose**

**dTDP-6-deoxy-L-glucose-lyxo-4-hexulose**

**dTDP-L-rhamnose**

**KR**

**DH**

**ER**

**DH (Dehydratase) domain**

**ER (enoylreductase) domain**

**KR (ketoreductase) domain**

**KS**

**AT**

**KS (ketosynthase) domain**

**M-AT**

**AT (acyl transferase) domain**

**– Malonyl-CoA**

**AT (acyl transferase) domain**

**– Methylmalonyl-CoA**

**TE**

**ACP (acyl carrier protein)**

**TE (thioesterase) domain**

**Inactive domain**


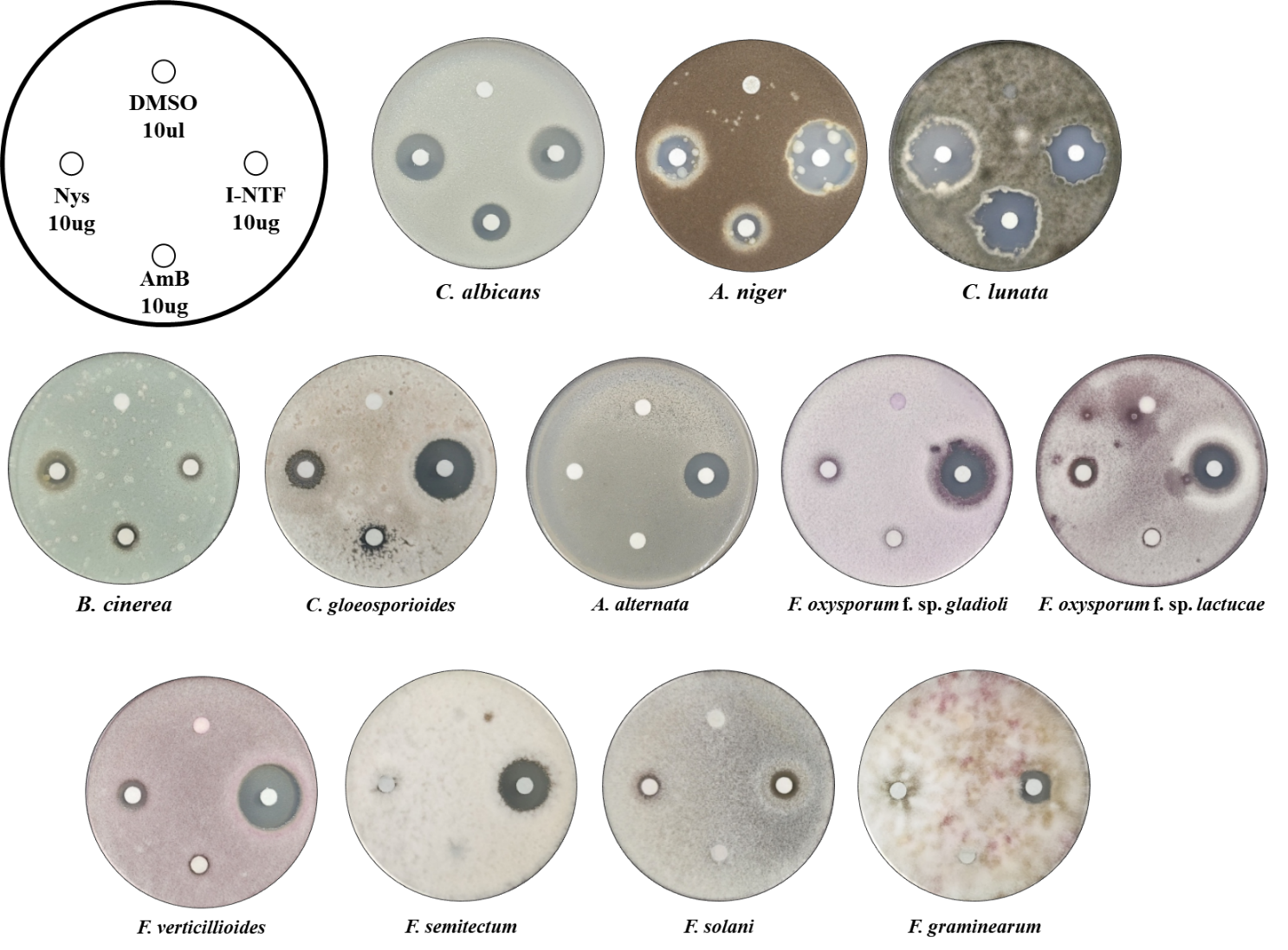


**Supplementary Figure S8.** *In vitro* antifungal activity of I-NTF using the RPMI-1640.The minimum inhibitory concentration (MIC) values were determined by measuring the minimum concentration that changed color to yellow.


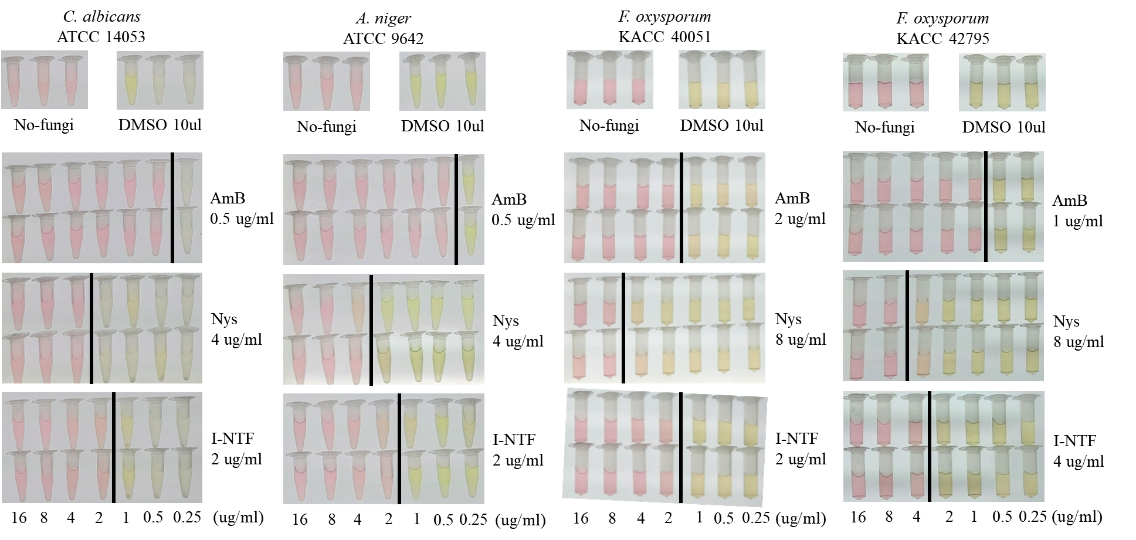


**
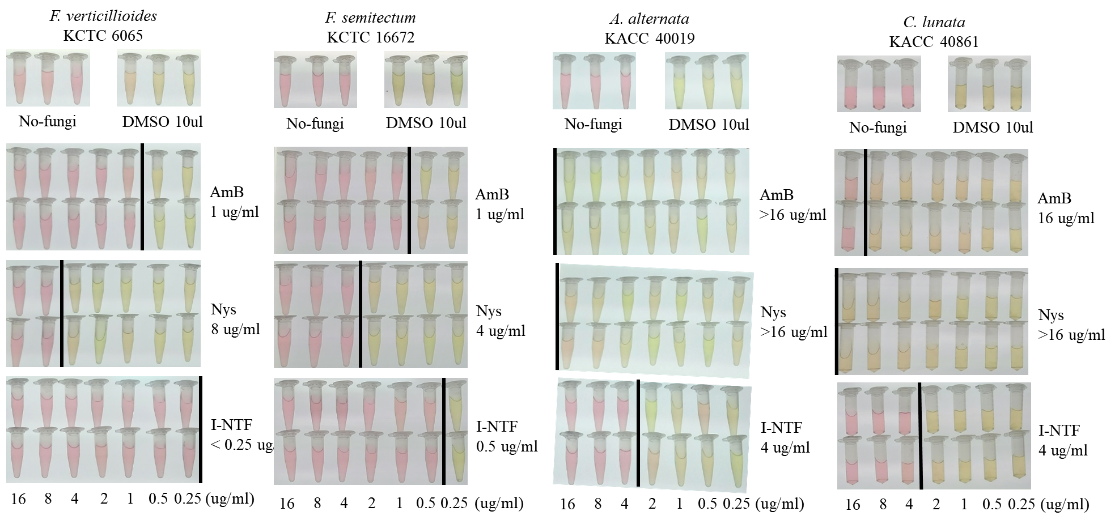
**

**
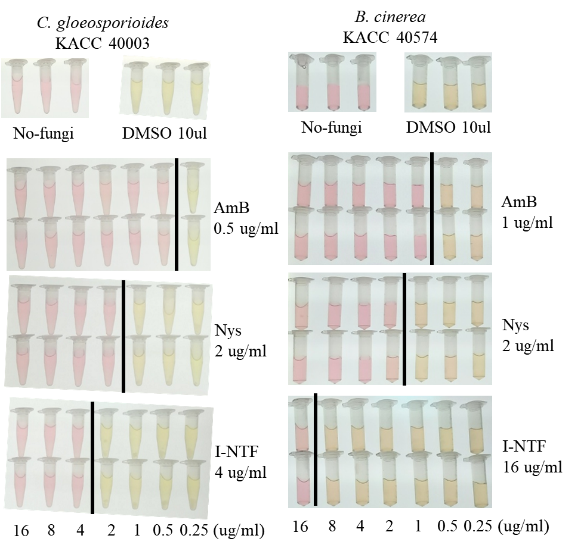
**

**Supplementary Table 1** Whole-genome sequencing of *S. rubrisoli* Inha501

1. Statistics of HiSeq rawdata.


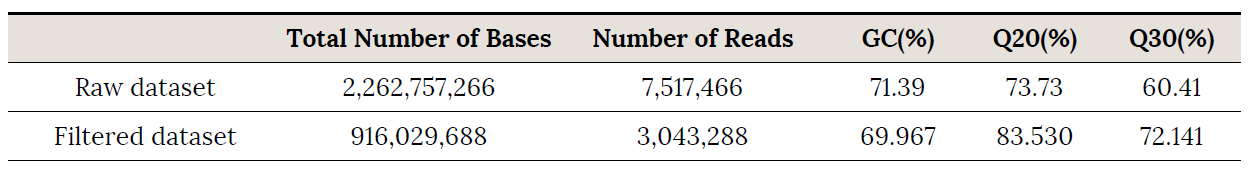


. Total Number of Bases: The Total number of bases sequenced.

. Number of Reads: The Total number of reads. In illumina paired-end data, read1 and read2 are added.

. GC(%) : GC content

. Q20(%): Ratio of bases that have phred quality score over 20.

. Q30(%): Ratio of bases that have phred quality score over 30.

1. Result of Assembly


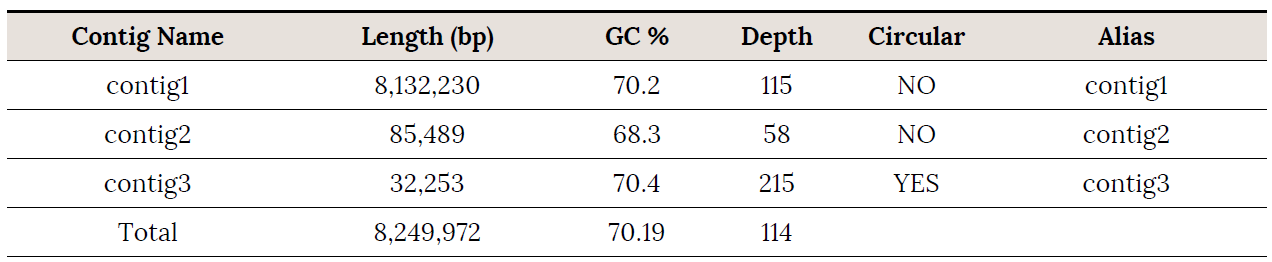


. Length(bp): The number of bases in each contig

. GC%: GC content

. Depth: The number of reads that overlap each contig

. Circular: 5’ end and 3’ end are connected.

. Alias: The alias is named based on the BLASTN(v2.6.0) result.

The following two conditions are used to create an alias:

a. Query cover 80% or more

b. Similarity between genome size

1. Result of Annotation


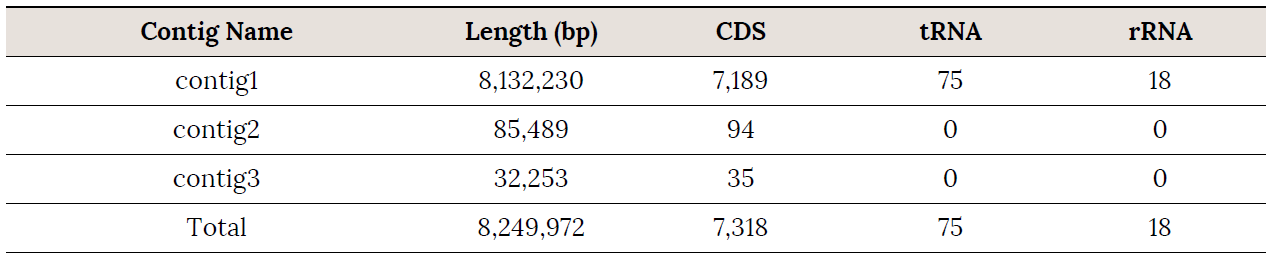


. Length(bp): The number of bases in each contig

. CDS: Coding Sequence

. tRNA: Transfer RNA, tRNA has triplet nucleotide sequence complementary to the triplet nucleotide coding sequences of messenger RNA (mRNA)

. rRNA: Ribosomal RNA, a molecular component of ribosome
